# List of Diseases from the 20th of June to the 20th of July; Being the Result of the Public and Private Practice of a Physician at the West End of the Town

**Published:** 1799-08

**Authors:** 


*List of Diseases from the 20th of June to the 20th of July; being the Result of the Public and Private Practice of a Physician at the West End of the Town.*

| ACUTE DISEASES. | No. of Cases. |
|---|---|
| Scarlatina Anginosa | 7 |
| Measles | 6 |
| Small-pox | 2 |
| Chicken-pox | 1 |
| Hooping-cough | 2 |
| Contagious malignant Fever | 3 |
| Acute Rheumatism | 7 |
| Catarrh | 5 |
| Ophthalmia | 3 |
| Inflammatory Sore-throat | 3 |
| Aphthous Sore-throat | 4 |
| Pneumonic Inflammation | 1 |
| Inflammation of the Bowels | 2 |
| Peritoneal Inflammation | 1 |
| Hæmorrhagy from the Bowels | 2 |
| Hæmorrhagy from the Lungs | 7 |
| Renal Hæmorrhagy | 1 |
| Epistaxis | 2 |
| Synochus, or Summer-fever | 6 |
| Child-bed and Milk-fever | 3 |
| Febrile Diseases of Infants | 9 |
| Hectica | 6 |

| CHRONIC DISEASES. | No. of Cases. |
|---|---|
| Cough and Dyspnœa | 32 |
| Pleurtic Stitches | 2 |
| Pulmonary Consumption | 7 |
| Chronic Rheumatism | 11 |
| Lumbago and Sciatica | 6 |
| Asthenia | 25 |
| Paralysis | 4 |
| Head-ach and Vertigo | 6 |
| Melancholia | 3 |
| Chorea | 1 |
| Hysteria | 1 |
| Diarrhœa & Bilious vomitting | 18 |
| Dyspepsia | 11 |
| Gastrodynia | 10 |
| Enterodynia | 6 |
| Devonshire Colic | 2 |
| Chlorosis and Amenorrhœa | 4 |
| Menorrhagia | 5 |
| Abortus | 2 |
| Fluor Albus | 5 |
| Dysury | 3 |
| Renal Ischuria | 7 |
| Enuresis | 1 |
| Tabes Mesenterica | 6 |
| Dropsy | 9 |
| Scrofula | 5 |
| Worms | 3 |
| Rickets | 3 |
| Jaundice | 4 |
| Scirrhus of the Liver | 1 |
| Scirrhus of the Uterus | 1 |
| Stone and Gravel | 3 |
| Itch and Prurigo | 7 |
| Lepra | 1 |
| Scaly Tetter | 2 |
| Lichen | 2 |
| Impetigo | 1 |
| Eczema | 1 |
| Ecthyma | 3 |
| Porrigo | 2 |
| Acne | 2 |

